# Toward improving respectful maternity care: a discrete choice experiment with rural women in northeast Nigeria

**DOI:** 10.1136/bmjgh-2019-002135

**Published:** 2020-03-05

**Authors:** Nasir Umar, Matthew Quaife, Josephine Exley, Abdulrahman Shuaibu, Zelee Hill, Tanya Marchant

**Affiliations:** 1 Department of Disease Control, London School of Hygiene & Tropical Medicine, London, UK; 2 Department of Global Health and Development, London School of Hygiene & Tropical Medicine, London, UK; 3 Department of Primary System Development, State Primary Health Care Development Agency, Gombe, Nigeria; 4 Institute for Global Health, University College London, London, UK

**Keywords:** health services research, maternal health, health economics, health policy, health systems

## Abstract

**Introduction:**

There is a limited understanding of the importance of respectful maternity care on utilisation of maternal and newborn health services. This study aimed to determine how specific hypothetical facility birth experience of care attributes influenced rural Nigerian women’s stated preferences for hypothetical place of delivery.

**Methods:**

Attributes were identified through a comprehensive review of the literature. These attributes and their respective levels were further investigated in a qualitative study. We then developed and implemented a cross-sectional discrete choice experiment with a random sample of 426 women who had facility-based childbirth to elicit their stated preferences for facility birth experience of care attributes. Women were asked to choose between two hypothetical health facilities or home birth for future delivery. Choice data were analysed using multinomial logit and mixed multinomial logit models.

**Results:**

Complete data for the discrete choice experiment were available for 425 of 426 women. The majority belonged to Fulani ethnic group (60%) and were married (95%). Almost half (45%) had no formal education. Parameter estimates were all of expected signs suggesting internal validity. The most important influence on choice of place of delivery was good health system condition, followed by absence of sexual abuse, then absence of physical and verbal abuse. Poor facility culture, including an unclean birth environment with no privacy and unclear user fee, was associated with the most disutility and had the most negative impact on preferences for facility-based childbirth.

**Conclusion:**

The likelihood of poor facility birth experiences had a significant impact on stated preferences for place of delivery among rural women in northeast Nigeria. The study findings further underline the important relationship between facility birth experience and utilisation. Achieving universal health coverage would require efforts toward addressing poor facility birth experiences and promoting respectful maternity care, to ensure women want to access the services available.

**Key questions**

**What is already known?**

- Poor facility birth experience is prevalent especially in countries where childbirth-related mortality is high, and facility-based childbirth is low.
- Little is known on how facility birth experience of care attributes influences women’s choice to deliver in a health facility or not.

**What are the new findings?**

- Women associated the most utility to good health system conditions, including having a qualified birth attendant, drugs and supplies and a clean and conducive birth environment.
- Poor facility culture, including an unclean birth environment with no privacy and unclear user fee, was the experience of care attribute associated with the most disutility.

**What do the new findings imply?**

- At this point in time, enhancing the delivery environment in Gombe would have the most positive impact on women preferences for facility-based childbirth.
- Interventions designed to improve respectful maternity care require greater understanding of women’s preferences.

## INTRODUCTION

High quality, facility-based care at the time of childbirth remains the main strategy to address the continuing high burden of maternal mortality and morbidity, especially in low-income and middle-income countries (LMICs).^1 2^ The provision of respectful and dignified care, including during childbirth, is an important component of quality of care and is the right of every woman.^3 4^ Any mistreatment during facility-based childbirth is a violation of women’s fundamental human right. It has also been highlighted as contributing to women not seek care at all or for subsequent deliveries, delaying seeking care, or discouraging others from delivering in health facilities.^3 5–7^ The recognition that experience of care during childbirth has an impact on the use of maternal and newborn health (MNH) services has made the elimination of mistreatment and the provision of respectful maternity care a public health issue of global importance.^3 8^

Advances in the methods for measuring experience of care have allowed the assessment of mistreatment during facility-based childbirth; with widespread mistreatment reported where it has been assessed.^9–13^ For example, in Nigeria, where a high burden of maternal mortality persists, and where the coverage of facility delivery remains relatively low, women have reported mistreatment due to improper examination and lack of supportive care,^3 13^ neglect or abandonment, lack of confidentiality, physical abuse, verbal abuse and mistreatment related to health system constraints.^11 14^ In a recent study in Gombe State, where we conducted this study, about two-thirds of women interviewed reported experiencing at least one form of mistreatment during their facility-based childbirth.^15^ Half of all women (50%) reported mistreatment related to health system conditions and constraints; for example, staff shortages, supply constraints, unconducive birth environment and perceptions of unreasonable requests by health workers. Similarly, almost half (46%) of all women reported mistreatment related to poor rapport between themselves and the healthcare provider; for example, denial of birth companion and not being allowed to eat, drink or move around during labour and childbirth. Reports of mistreatment related to sexual abuse, stigma and discrimination were uncommon.^15^

Being able to define and assess mistreatment provides the opportunity to design effective interventions across a range of attributes including professional standards of care, rapport between women and providers, health system conditions and constraints, physical abuse, verbal abuse, sexual abuse and discrimination, to improve facility birth experience.^3 9 10^ However, to date there is limited evidence on how these different attributes of experience of care during childbirth interact to influence women’s choice of whether or not to deliver in a health facility. This study investigates how these attributes influence a woman’s choice for a place of delivery in Gombe State, northeast Nigeria. Through a discrete choice experiment (DCE), we aim to determine the relative value (utility) rural women in northeast Nigeria place on a range of attributes of experience of care during facility-based birth.

## Methods

### Study context

This study was conducted in Gombe state in northeast Nigeria. MNH indices are suboptimal for Nigeria as a whole but there is also considerable regional variation, with the northwest and northeast regions having lower utilisation of healthcare compared with southern regions of the country.^16 17^ This is true of Gombe State which, relative to national estimates, has lower coverage of at least four antenatal care visits (44% vs 57%), lower coverage of facility delivery (28% vs 39%) and higher infant mortality rate (90/1000 live births vs 70/1000 live births).^17^ Almost 98% of formal health services in Gombe State are provided through government at three levels—primary, secondary and tertiary levels.^18 19^ Primary health services are delivered through dispensaries, health posts, health centres and primary health centres, and secondary services are provided through the state specialist hospital and general hospitals, which also serve as referral centres. Tertiary services are provided through the Federal medical centre.^18 19^ The majority of healthcare workers in Gombe State are lower cadre for example, Community Health Extension Workers (CHEWS), Junior CHEWS and Health officers.^20 21^ Skilled healthcare providers including medical doctors and nurses/midwives constitute only 4% and 27% of the health workforce, respectively.^20 21^ The majority of women deliver in primary healthcare facilities, attended to by the lower cadre healthcare workers.^20^

### Study design

#### Identification of attributes and levels

In healthcare, patients or clients often have strong preferences for treatment and health service options, and these can affect service utilisation.^22 23^ In this study, conducted in March 2018, we used DCE to elicit women’s preferences for a facility-based birth based on different attribute levels presented in table 1. DCEs are health economic tools, widely used to understand user preferences in healthcare.^24^ DCEs ask respondents to choose their preferred service from a set of hypothetical alternatives over several choice tasks. Studying how respondents choose across repeated scenarios allows researchers to quantitatively elicit the key drivers of decision making.^25–28^

**Table 1:** Attributes used in discrete choice experiment on respectful maternity care attributes influencing women’s stated preferences for facility-based childbirth

Attributes	Attribute levels
Failure to meet standards of care	Lack of informed consent and confidentiality: birth attendant may not ask for your permission before performing any medical examination or procedure and may discuss your personal information openly with others.
	Physical examinations and procedures: birth attendant may not give you pain relief as necessary (eg, during examination) or for the stitching of episiotomy.
	Neglect and abandonment: birth attendant may leave you unattended, you may deliver without assistance.
	Meet professional standards of care: Birth attendant will take care of you throughout labour and delivery, will ask for your consent, provide pain relief when needed.
Poor rapport with providers	Poor communication: birth attendant may not explain what will happen to you or your baby and may not encourage you to ask questions or answer your questions.
	Lack of supportive care: birth attendant will not empathise or show genuine interest in your well-being.
	Loss of autonomy: birth attendant will not allow a companion to stay with you and will not allow you to eat, drink, move about or choose your preferred birth position.
	Good rapport with providers: Birth attendant will receive you with open arms, comfort and encourage you, respond to you in friendly way, allow you to have a companion and to choose your preferred birth position.
Health system constraints	Staffing constraints: qualified birth attendant not available to assist with your delivery.
	Drugs and supply constraints: drugs and supplies needed for delivery not available.
	Poor facility culture: birth environment unclean, smelly, with mosquitoes, with no privacy, user fee not clear.
	Good health system conditions: qualified birth attendant present to assist you and drugs and supplies needed for delivery available, clean and conducive birth environment.
Physical and verbal abuse	Physical and verbal abuse: birth attendant may hit, slap or put restraints on you, shout or insult you.
	No physical and verbal abuse: birth attendant will not hit, slap or put restraints on you and will not shout or insult you.
Sexual abuse	Sexual abuse: birth attendant may touch your body parts or private parts inappropriately.
	No sexual abuse: birth attendant will not touch your body parts or private inappropriately.
Stigma and discrimination	Discrimination: birth attendant may discriminate against you because you are poor, from a village, not educated or because of your religion, tribe or disease condition.
	No discrimination: birth attendant will not discriminate against you because you are poor, from a village, not educated or because of your religion, tribe or disease condition.

Attributes and attribute levels derived from literature review,^3^ and revised based on qualitative findings.

The first step in designing a DCE is to select the key service attributes—or characteristics—which may be important to users. To do this, we conducted a comprehensive review of the literature to identify attributes of respectful maternity care. Data bases searched included PubMed, Google Scholar, EMBASE, CINAHL and EBSCO. We further searched the reference list of the identified articles and reached out to experts in MNH to identify additional literature. From the results of the review, we selected the revised typology of mistreatment by Bohren *et al*,^3^ based on a systematic review of 65 studies from 34 countries. The typology builds on earlier work of Bowser and Hill^29^ and revised the dimensions of mistreatment to include seven domains: physical abuse, sexual abuse, verbal abuse, stigma and discrimination, failure to meet professional standards of care, poor rapport between women and health providers and health system conditions and constraints. We used the typology to design a qualitative study to investigate the relevance of the dimensions in the study setting, and to derive attribute levels by investigating how the different dimensions manifest for each attribute.

The qualitative study included in-depth interviews with 31 women and four focus groups with 32 women (eight women per focus group). The qualitative study participants were women who had recently delivered in a health facility, purposively sampled from the communities. The qualitative study was conducted in December 2017. The qualitative data were analysed using thematic content analysis, with a manifest approach,^30^ in which the data analysis focused on what women said about their experience during labour and delivery.

In-depth interviews and focus groups, alongside the literature review findings, informed our selection of six attributes and a total of 18 attribute levels relevant to the context (three attributes of four levels each and three attributes of two levels), these are highlighted in table 1. Further, the qualitative study provided us with locally appropriate expressions and language translation, enhancing respondents ease of comprehension.^31^

### Experimental design and construction of choice sets

From the number of attributes and attribute levels decided, a full factorial design would have consisted of 729 (3^4^×3^2^) possible alternatives—too many for a survey, and tedious for the respondents to handle.^27 32^ Therefore, we developed 16 choice sets based on a fractional factorial orthogonal main effects design from a design catalogue, that ensured the inclusion of levels proportionally (level balance) with no correlation between levels of different attributes (orthogonal).^32^ We constructed an unlabelled choice experiment of three choice alternatives, with each set consisting of two unlabelled facility alternatives and home delivery, an example is highlighted in table 2. We decided to include a home delivery option to avoid bias in estimating parameters in a forced choice design.^33^

**Table 2:** Example of discrete choice experiment choice task as shown to women who had a facility-based childbirth

We would like you to imagine you are deciding where you are going to deliver your next baby, tell us which of the options in each scenario you will prefer to go to (or not) for your delivery. There are no right or wrong answers, we are only interested in knowing what is important to you regarding facility delivery. Please tick your preferred choice.	
Hospital A	Hospital B
Birth attendant may not ask for your permission before performing any medical examination or procedure and may discuss your personal information openly with others.	Birth attendant may not give you pain relief as necessary (eg, during examination) or for the stitching of episiotomy.
Birth attendant may not explain what will happen to you or your baby and may not encourage you to ask questions or answer your questions.	Birth attendant will not empathise or show genuine interest in your well-being.
Qualified birth attendant not available to assist with your delivery.	Drugs and supplies needed for delivery not available.
Birth attendant may hit, slap or put restraints on you, shout or insult you.	Birth attendant will not hit, slap or put restraints on you and will not shout or insult you.
Birth attendant may touch your body parts or private parts inappropriately.	Birth attendant will not touch your body parts or private inappropriately.
Birth attendant may discriminate against you because you are poor, from a village, not educated or because of your religion, tribe or disease condition.	Birth attendant will not discriminate against you because you are poor, from a village, not educated or because of your religion, tribe or disease condition.
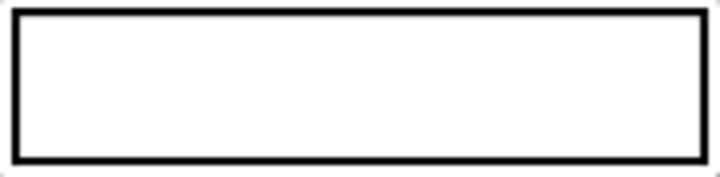 ­	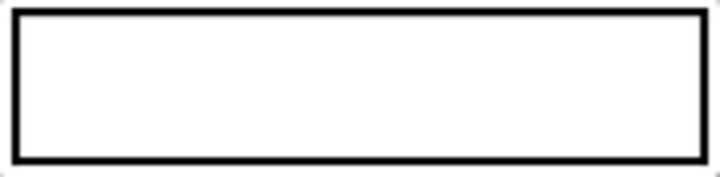
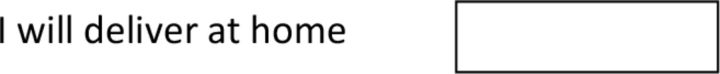 ­	

The choice sets were reviewed and validated for content in collaboration with a group of health workers (doctors, nurses and midwives) working in Gombe, followed by a pilot test with 40 women with similar characteristics as the target sample (women with recent facility-based childbirth). None of the women that took part in the pilot participated in the main study. The pilot exercise involved completing the DCE and answering questions afterwards regarding the exercise, including clarity of instructions, understanding and relevance of the choice sets and ease in answering. Following the pilot, minor modifications were made which included small changes to the wording used to describe the attribute levels, and to introduce the choice sets. For example, participants from the pilot suggested we use ‘hospital’ rather than ‘health facility’ when describing the choice sets. The final DCE tool was incorporated into a larger study instrument consisting of questions on sociodemographic information, and experience of care during institutional delivery. The final questionnaire was programmed in CSPro.^34^

### DCE study sample

The final DCE was nested within an ongoing measurement, learning and evaluation project in Gombe State.^35^ As part of that project, a total of 1889 birth observations were carried out in ten primary health facilities across three time points: June 2016, March 2017 and August 2017.^36^ The health facilities were spread across six of the 11 local government areas in Gombe State. Six of the health facilities were located in urban settings and four were located in rural settings. Subsequently, in August 2018, a simple random sample of 450 observed women was taken and these women were followed up at home to ask what they recalled about their facility birth experience. Of the 450 eligible and selected women, 426 (95%) women were successfully interviewed in March 2018 at their homes, while 24 (5%) of the eligible women selected could not be reached or were unable to participate. According to commonly used rules of thumb for DCE sample size calculation, the sample size of 426 women was enough to guarantee precision in the estimation of all model parameters.^37^ Orme posit that the sample size *N* required for main effects depends on the number of choice task (*t*), the number of alternatives (*a*) and the number of cells (*c*), according to the following equation *N*>500 *c*/(*t*×*a*).^37 38^ Based on this equation, an approximate sample of 56 participants would have been sufficient to model our preference data. Lancsar and Louviere suggested that a sample of 20 respondents per questionnaire version as adequate to estimate reliable DCE models.^39^ However, we recruited much larger sample to allow for more variability between respondents and to allow for other post hoc analysis.

### Data collection

Data were collected using personal digital assistants, and interviews took about an hour to complete. Before data collection, data collectors and supervisors received 5 days of training on data collection and the study tools.

### Model specification

The discrete choice data were analysed based on the random utility model.^25^ We specified our analytical model around a utility maximising framework. This assumes, given alternatives to choose from, a respondent *i* (*i*=1, …, *N*) will choose the one alternative that yields the maximum utility among the choice bundle (*j*=1, 2, 3, …) at the moment of choice. The utility of the respondent is defined by a deterministic or observable component and a random error component:

(1) $$U_{ij}=v_{ij}+\varepsilon_{ij}$$

Where *U_ij_* represents the utility of respondent,*v_ij_* the observable component and *ε_ij_* the random error term with standard statistical properties. Following from equation (1) the probability of a respondent selecting a specified place of delivery is modelled. The probability of a choosing a place of delivery is determined by the indirect utility function for the respondent *i* from choice *j* in choice set *s*, assuming this is linear and additive and of the form:

(2) $$V_{ijs}=X_{ijs}\beta +\varepsilon_{ijs}$$

Where *V*_*ijs*_ represent the utility derived from a choice, and *X*_*ijs*_*β* the utility component and *ε*_*ijs*_ as the random component. The vector *X_ijs_* is specified below, where *β*_1-6_ represent the design attributes of the choice experiment and *β*_0_ the constant.

(3) $$\begin{array}{ll} X_{ijs}\beta_{j}= & \beta_{0}+\beta_{1}{failure\_to\_meet\_standard\_of\_care}_{j}+\\ & \beta_{2}{poor\_rapport\_with\_provider}_{j}+\beta_{3}health\\ & {\_system\_constraints}_{j}+\beta_{4}physical\_\&\\ & {\_verbal\_abuse}_{j}+\beta_{5}{sexual\_abuse}_{j}\\ & +\beta_{6}stigma\_{\_discrimination}_{j} \end{array}$$

### Data analysis and model estimation

We analysed the DCE data using STATA V.15. We conducted a dominance test of internal validity, presenting an additional choice set to all the respondents where one of the hypothetical health facilities is more favourable, but do not exclude participants if they fail this test.^40^ We first estimated standard multinomial logit model (MNL) (online supplementary table S1) to provide a benchmark for more detailed analysis, and which were used as starting values for estimating a mixed multinomial logit model (MMNL) (online supplementary table S2). Further, we fitted MMNLs to the DCE data from rural women investigated, using 500 Halton draws. We used the MMNL to avoid restrictions due to the independence of irrelevant alternatives assumption required to interpret findings from the MNL.^41 42^

10.1136/bmjgh-2019-002135.supp1 Supplementary data

When estimating MMNLs all parameter estimates may be treated as random, and in a model where more than one parameter or all parameters are estimated as random, there is no requirement that the distribution be the same.^43^ McFadden and Train have shown that mixed multinomial logit does not embody any theoretical restrictions on the distribution of preferences or the choice model.^44^ Also, with mixed multinomial logit, we can accurately approximate any choice model, with any distribution of preferences.^44^ Therefore, all attributes levels were effects coded, specified as random components and a multivariate normal distribution—a generalisation of the one-dimensional normal distribution to more than one variable was assumed. Assuming a multivariate normal distribution to represent the distribution of a multivariate random variable that is made up of multiple random variables that can be correlated with each other was a rational choice because this allows for correlation which introduces error dependence across the alternatives in each choice situation.^45 46^ Furthermore, assuming a random effect to be respondent specific induces correlation across choice situations, thus accounting for the dependence structure in unobserved utility among the repeated choices of a respondent due to the panel structure of the data.^45^ Findings are reported below in line with Strengthening the Reporting of Observational Studies in Epidemiology statement.^47^

### Preference heterogeneity

An important output from the main effects MMNL I estimation is the SD associated with each parameter estimate, which indicate the distribution about the corresponding mean preference weight, also the preference variability among women around the mean. Some of the differences between the parameter estimates in the multinomial logit and MMNLs further indicate that preferences vary among women in Gombe.

Respondents characteristics are constant across alternatives for example, a woman’s ethnicity does not change because she is considering delivering in a health facility as opposed to home delivery.^43^ Respondents characteristics are likely to influence their choice decisions, but they are not part of the attribute description of alternatives and not a direct source of utility.^43^ One way to predict how respondents’ characteristics influenced their choices is to extend equation (2) to allow attribute weights to vary with respondent characteristics, through the inclusion of interaction terms between attribute and individual characteristics.^48^

Therefore, to understand the preference (taste) variability among women the MMNL (model I) was extended with interaction terms between attribute levels with significant SD and women sociodemographic characteristics likely to influence women’s behaviours or predisposition to mistreatment, as highlighted in equation (4) below (and MMNL model II). This approach often leads to models fit improvement. It is also easy to interpret the parameters related to covariates in a relative sense both within and between alternatives—holding all else equal.^43 48^ Revelt and Train have shown that entering demographics into the MMNL itself is a more direct and accessible way to hypothesis testing, as such, this approach has been widely used in DCE studies.^49 50^

(4) $$\begin{array}{ll} X_{ijs}\beta_{j} & =\beta_{0}+\beta_{1}failure\_to\_meet\_standard\_of\_care_{j}\\ & +\beta_{2}poor\_rapport\_with\_provider_{j}+\beta_{3}\\ & health\_system\_constraints_{j}+\beta_{4}\\ & physial\_\&\_verbal\_abuse_{j}+\beta_{5}sexual\_abuse_{j}+\\ & \beta_{6}stigma\_\&\_discrimination_{j}+\beta_{7}female*\\ & neglect\_abandonment_{j}\;......+\beta_{26}upperSES*\\ & no\_sexual\_abuse_{j} \end{array}$$

Where *β*_1-6_ represent the design attributes of the choice experiment and *β*_7–26_ the parameters for the interaction terms that were introduced for the women sociodemographic characteristics variables. The sociodemographic characteristics used included ethnicity coded (0=others, 1=Fulani), age coded (0= ≤29 years, 1= ≥30 years), education coded (0=low-level education, 1=high-level education) and socioeconomic status (SES) coded (0=low SES (lower 60%), 1=upper SES (upper 40%).

## Results

### Demographic characteristics of women

The final sample used in the analysis comprised 425 respondents; one case was dropped due to incomplete data. Descriptive statistics for the study sample are displayed in table 3. The sample was young, with 60% of the respondents being between the ages of 20 and 29 years, and just 3% were between the ages of 40 and 49 years. The majority belonged to the Fulani ethnic group 60%, followed by Hausa. Respondents were mostly married (95%) and Muslims (98%). Almost half (45%) had no formal education. Only about 7% failed the dominance test by choosing to deliver in a less favourable hypothetical health facility, suggesting acceptable internal validity.^51 51 52^

**Table 3:** Demographic characteristics of women in discrete choice experiment on facility birth experience of care attributes influencing women’s stated preferences for facility-based childbirth

Characteristics and delivery context	n=425 (%)
Age	
<20 years	11
20–29 years	60
30–39 years	26
40–49 years	3
Ethnicity	
Fulani	60
Hausa	17
Kanuri	7
Others	16
Religion	
Christian	2
Muslim	98
Marital status	
Married	95
Single/widowed	5
Education level	
None	45
Primary	23
Secondary and postsecondary	32
Parity	
Primigravida	0
Multigravida	100
Period of birth	
Day time (07:00 to 19:59)	55
Night time (20:00 to 06:59)	45
Day of delivery	
Weekdays	78
Weekend	22

### Model estimates of preferences for attribute-levels of respectful maternity care

**Figure 1:**
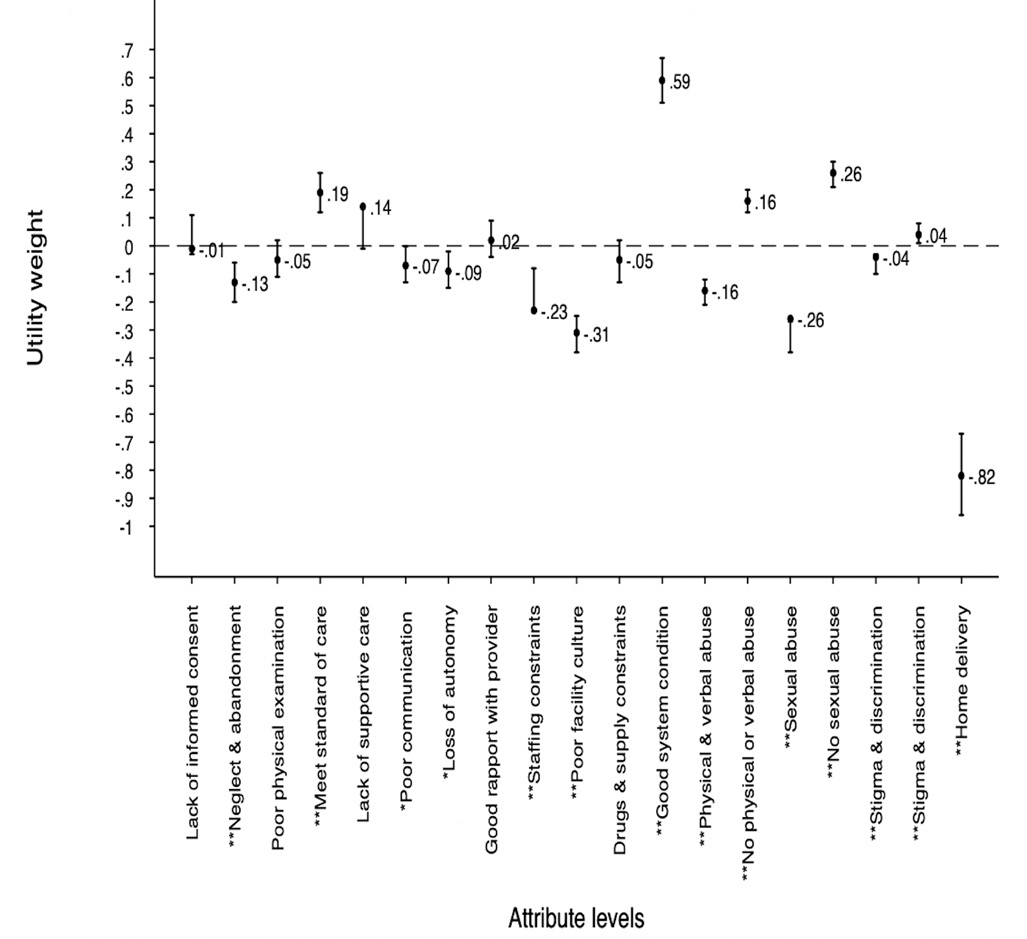
Positive values indicate positive effect on preference, negative values indicate negative effect on preferences. **Significance level at 1% and * significance level 5%.

Figure 1 and online supplementary table S2 highlight the utility parameter estimates from main effects MMNL for preferences for facility birth experience of care attributes levels. The positive sign of the estimated coefficients indicates that the attribute level has a positive effect on preferences for facility-based childbirth. Conversely, a negative coefficient indicates a negative effect on preference. The estimated coefficients all followed anticipated direction of effect; implying women derived higher levels of utility from attribute levels which were considered better ex ante.

In the main effects model (model I), most estimated coefficients were statistically significant. At least one attribute level was significant in each of the attributes included in the study, indicating all attributes contributed to the decisions made by women when stating their preferences for a place of delivery.

The most important attribute level that influenced the likelihood of women choosing a facility-based childbirth was good health system condition, including having qualified birth attendant present during childbirth, drugs and supplies needed for delivery available, clean and conducive birth environment (coefficient: 0.59), followed by absence of sexual abuse (coefficient: 0.26). Absence of physical and verbal abuse during labour and delivery was the third most preferred attribute level among women (coefficient: 0.16) (figure 1 and online supplementary table S2).

For rural women in Gombe, poor facility culture including unclean birth environment, with no privacy and unclear user fee was associated with the most disutility, therefore, the most negative impact on preferences for facility-based childbirth (coefficient: −0.31), closely followed by sexual abuse (coefficient: −0.26) and staffing constraints (coefficient: −0.23). The likelihood of the respondents being discriminated against because of a disease condition or because they were poor, not educated or from a village had the least influence on their choice of place of delivery. Home delivery was also associated negative utility (figure 1 and online supplementary table S2).

### Preference heterogeneity

The influence of sociodemographic characteristics on preferences highlighted in online supplementary table S2 (interaction model II) suggested variation in preferences for some facility birth experience of care attributes among rural women in Gombe. Women of lower SES were more likely to choose to deliver in a hypothetical health facility where they are less likely to be neglected or abandoned. Absence of mistreatment related to sexual abuse had less effect on preference for place of delivery among women with low education (ie, no formal education or only primary school education) and those that identified as non-Fulani by ethnicity. However, the preference variability observed could not be explained by observed characteristics of women. Considerable variation (indicated by SD in MMNL II) still remained even after including ethnicity, age, education and SES variables.

## Discussion

In this study, we explored preferences for facility birth experience of care attributes among rural women with recent facility-based childbirth in northeast Nigeria. An in-depth understanding of how women value different facility birth experience of care attributes is of global interest since it could provide the foundation for developing and designing appropriate interventions that could improve utilisation. Our findings suggest that hypothetical facility birth experiences are influential in women’s decision making about place of delivery.

Based on the magnitude and direction of the estimated attribute-level coefficients, women associated varying degrees of utility and disutility to hypothetical facility birth experience of care attributes levels. We found that women associated the most utility to good health system conditions, including having a qualified birth attendant, drugs and supplies, and a clean and conducive birth environment. Women expressed strong preference to deliver in a health facility where they were less likely to be sexually, physically or verbally abused, as absence of sexual abuse and absence of physical and verbal abuse during labour and delivery were the second and third most important attribute levels for rural women in this study. But the experience of care attribute they associated the most disutility to was poor facility culture, including an unclean birth environment with no privacy and unclear user fee.

Our findings are consistent with studies from Ethiopia, Ghana, Kenya, Nigeria and the Philippines which have reported a strong association between improvements in structural quality and the availability of commodities with a substantial increase in health facility utilisation.^53 54^ Interestingly, the utility associated with drug and supply constraints was negative but did not reach statistical significance, perhaps a reflection of women in Gombe having coping strategies for persistent shortages of drugs and supply by sourcing these outside of the health system, rather than an indication of not caring about drugs and supply constraints.^15^

Studies in many LMICs have also reported a widespread prevalence of physical, verbal and sexual abuse during facility-based birth.^3 13 55^ Women have reported incidences of being treated differently due to their SES or disease condition,^3 13 55^ and incidences of being denied their preferred birth position, or not allowed to eat, drink or move.^3 55^ These negative experiences during facility-based birth have been found to undermine women’s trust in the health system, and negatively impacted their subsequent decision to deliver in the health facility.^56–58^ In an earlier study in Gombe State, only 3% of women reported mistreatment related to physical abuse, and less than 1% reported mistreatment related to sexual abuse during labour and delivery.^15^ Nonetheless, we found that absence of physical abuse and absence of sexual abuse influenced women’s hypothetical choice for place of delivery. Our findings suggest that the low prevalence of a particular negative facility birth experience, for example, sexual abuse or physical abuse does not negate its impact.

We found few of the women’s observable characteristics could explain the variability in preferences for the attribute levels. These included ethnicity, SES and level of education. Women with low education or women of low SES were more concerned with the likelihood of experiencing certain forms of mistreatment when making a choice for a place of delivery, than women with more years of education or high SES. It is probable that differential experiences of women from different communities with their respective health facilities influenced the value they associated with attributes where heterogeneity was observed. Kruk *et al* argued that respondents draw on their experiences or that of their friends or families when conducting DCE exercises.^54^ Variability in preference for facility birth experience of care attributes has been observed elsewhere. A study in urban Beirut found that women preferred their husbands to accompany them during labour and delivery, while women in the rural areas preferred to be cared for by healthcare providers alone.^59^ Understanding how preferences vary between women could improve interventions by making them more responsive and oriented towards patient-centred care.

This study had strengths and limitations. The results represent the predominantly Fulani, Muslim, married study participants who accessed the study health centres. These women had their last birth in a health facility and had therefore experienced facility-based respectful maternity care, or the lack of it, so our findings may not apply to women with no facility delivery experience. Further, in this context and other similar settings, the decision to deliver in a health facility may not rest exclusively with women, as household heads, and in-laws may also have a say. In this study we used qualitative methods to develop and refine our hypothetical scenarios, but still they may not have reflected real-world options for place of delivery. Nonetheless, in previous studies, DCEs has been shown to predict real-world choices with a reasonable degree of sensitivity and external validity.^51^

### Implications for policy and research

Access, cost, sociocultural barriers and other contextual issues continue to be important determinants of utilisation of MNH services in LMICs.^3 57 58 60^ Here we underline the additional importance of respectful maternity care as a determinant of women’s decision to deliver in a health facility. The renewed global drive to eliminate mistreatment and improve respectful maternity care as part of the quality of care in health facilities has given the evaluation of the experience of care a new impetus.^3 4^ Strategies toward improving respectful maternity care could be made more efficient with additional understanding of the utility and disutility women associate with attributes of care. For example, evidence on the extent to which absence of physical abuse during labour or having a birth companion mattered to women could help decision-makers to develop strategies that are more context-appropriate and relevant to women, rather than transplanting interventions because they worked elsewhere.

This study reinforces the importance that women attribute to both structural and process quality of care. In a recent study in Gombe State, most women in the qualitative study suggested that the possibility of receiving drugs, injections and skilled assistance during childbirth was a sufficient motivation to deliver in a health facility again.^15^ However, women also described the various coping mechanisms they applied in anticipation of mistreatment, for example, bypassing the facility closest to their home^61^ or delaying going to the health facility to limit how much time they spent in an unconducive birth environment. Such coping mechanisms could have several negative implications, for example, on birth outcomes and on increased out of pocket expenditure for health due to increased transport costs. Catastrophic health expenditure has been increasing worldwide, and more people around the world are pushed into extreme poverty yearly due to out-of-pocket expenses.^62^ Nigeria’s drive towards achieving the sustainable development goals must include addressing causes that increase out of pocket spending for health and perpetuates poverty.

To achieve Universal Health Coverage, the Nigerian government is planning to build, upgrade or refurbish about 10 000 health facilities across the country.^63^ Our study has shown that women prioritise good health system conditions in their decision making and these new health facilities need to be adequately staffed as well as equipped and stocked with essential supplies. Otherwise, women are very likely to continue bypassing facilities. As part of these strategies, improving the enabling environment for health staff, including building their capacity to deliver respectful maternity care, could improve interactions between providers and women.^63 64^

Inevitably, addressing these problems also means more resources, difficult to achieve in a low resource setting. Hence, there is a need to rationally consider effective trade-offs when designing interventions to improve respectful maternity care, trade-offs that are likely to be context specific and may change overtime.^65^ A strategy of focusing first on addressing mistreatment in areas where most benefits can be obtained quickly while keeping an eye on the whole picture is worth considering.

## Conclusions

In this study we used a DCE to explore how hypothetical facility birth experience influenced rural woman’s choice for place of delivery in northeast Nigeria. Women associated the most utility to good health system conditions, including having a qualified birth attendant, drugs and supplies and a clean and conducive birth environment. Conversely, respondents were less likely to choose to deliver in a hypothetical health facility where the health facility culture was poor, including unclean birth environment with no privacy and unclear user fee. The results further underline the important relationship between facility birth experience and utilisation. The study findings suggest achieving universal health coverage will require not only that health services are available, but are available to a sufficient quality that women would want to access them.
