# Supplementary material for: Toward improving respectful maternity care: a discrete choice experiment with rural women in northeast Nigeria
Source: BMJ Glob Health. 2020 Mar 5;5(3):e002135. doi: 10.1136/bmjgh-2019-002135 (PMC7059545; doi:10.1136/bmjgh-2019-002135)
Supplement: Supplementary data [file bmjgh-2019-002135supp001.pdf]

Supplementary:

S1. Multinomial logit model results for experience of care attributes influencing women's stated preferences for facility-based childbirth

Attributes & attribute levels	Mean β(SE)
**Failure to meet standard of care**: Lack of informed consent (Ref)	-0.003(0.03)
Neglect, abandonment	-0.130(0.03)[a]
Poor physical examination & procedure	-0.028(0.03)
Meet standard of care	0.155(0.03)[a]
**Poor rapport with provider**: Lack of supportive care (Ref)	-0.120(0.03)[c]
Poor communication	-0.054(0.03)[c]
Loss of autonomy	-0.076(0.03)[b]
Good rapport with provider	0.010(0.03)
**Health system constraints**: Staffing constraints (Ref)	0.150(0.04)[a]
Poor facility culture	-0.286(0.03)[a]
Drugs & supply constraints	-0.045(0.03)
Good health system condition	0.481(0.03)[a]
**Physical & verbal abuse**: Physical & verbal abuse (Ref)	-0.138(0.02)[a]
No physical or verbal abuse	0.138(0.02)[a]
**Sexual abuse**: Sexual abuse (Ref)	-0.205(0.02)[a]
No sexual abuse	0.205(0.02)[a]
**Stigma & discrimination**: Stigma & discrimination (Ref)	-0.039(0.02)[a]
No stigma & discrimination	0.039(0.02)[a]
**Home delivery**: No facility birth benefits or mistreatment	-0.312 (0.02)[a]
Log likelihood	-6445.18
AIC	12916.36

[a-c] Significance at 1% (a), 5% (b), 10% (c) levels

S2. Mixed logit model results for discrete choice experiment on experience of care attributes influencing women's stated preferences for facility-based childbirth

	Main effects model (I)	Interaction model (II)
Attributes & attribute levels	Mean β(SE)	Mean β(SE)
**Failure to meet standard of care**: Lack of informed consent (Ref)	-0.01(0.04)	0.28(0.07)[b]
Neglect & abandonment	-0.13(0.04)[a]	-0.30(0.10)[b]
Poor physical examination & procedure	-0.05(0.03)	0.08(0.07)
Meet standard of care	0.19(0.03)[a]	0.10(0.09)
**Poor rapport with provider**: Lack of supportive care (Ref)	0.14(0.04)	0.14(0.04)[c]
Poor communication	-0.07(0.03)[b]	-0.07(0.03)[b]
Loss of autonomy	-0.09(0.03)[b]	-0.09(0.03)[b]
Good rapport with provider	0.02(0.03)	0.02(0.03)
**Health system constraints**: Staffing constraints (Ref)	-0.23(0.04)[a]	-0.25(0.05)[b]
Poor facility culture	-0.31(0.03)[a]	-0.33(0.05)[a]
Drugs & supply constraints	-0.05(0.04)	-0.07(0.05)
Good health system condition	0.59(0.04)[a]	0.65(0.12)[a]
**Physical & verbal abuse**: Physical & verbal abuse (Ref)	-0.16(0.02)[a]	-0.21(0.04)[a]
No physical or verbal abuse	0.16(0.02)[a]	0.21(0.06)[a]
**Sexual abuse**: Sexual abuse (Ref)	-0.26(0.03)[a]	-0.16(0.07)[b]
No sexual abuse	0.26(0.03)[a]	0.16(0.07)[b]
**Stigma & discrimination**: Stigma & discrimination (Ref)	-0.04(0.02)[a]	-0.04(0.02)[a]
No stigma & discrimination	0.04(0.02)[a]	0.04(0.02)[a]
**Home delivery**: No facility birth benefits or mistreatment	-0.82(0.07)[a]	-0.76(0.11)[a]
**Sociodemographic interactions**		
Fulani* Neglect & abandonment		0.14(0.10)
Fulani* Meet standard of care		0.15(0.10)
Fulani*Good health system condition		0.02(0.11)
Fulani* No physical or verbal abuse		-0.04(0.08)
Fulani* No sexual abuse		0.16(0.09)[c]
>=30yrs* Neglect, abandonment		0.01(0.11)
>=30yrs* Meet standard of care		0.09(0.11)
>=30yrs* Good health system condition		-0.16(0.12)
>=30yrs* No physical or verbal abuse		-0.03(0.08)
>=30yrs* No sexual abuse		0.10(0.10)
High-level education* Neglect, abandonment		0.15(0.11)
High-level education* Meet standard of care		0.07(0.11)
High-level education*Good health system condition		0.03(0.12)
High-level education* No physical or verbal abuse		-0.02(0.09)
High-level education* No sexual abuse		0.18(0.10)[c]
Upper-SES* Neglect, abandonment		0.21(0.10)[b]
Upper-SES* Meet standard of care		0.08(0.11)
Upper-SES* Good health system condition		0.03(0.12)
Upper-SES* No physical or verbal abuse		-0.10(0.08)
Upper-SES* No sexual abuse		-0.09(0.10)
Log likelihood	-5701.20	-5691.07
AIC	11454.41	11474.14

[a-c] Significance at 1% (a), 5% (b), 10% (c) levels
Home delivery: opt-out option for not choosing to deliver in a health facility
Model I: main effects mixed multinomial logit model estimating preferences for attribute levels
Model II: mixed multinomial logit model of interactions with sociodemographic characteristics - ethnicity (others vs Fulani), age (>=30years vs <=29 years), Education (high-level vs low-level) and socioeconomic status (SES) (upper-SES (upper 40%) vs low-SES (lower 60%)).
